# The Thalidomide-Binding Domain of Cereblon Defines the CULT Domain Family and Is a New Member of the β-Tent Fold

**DOI:** 10.1371/journal.pcbi.1004023

**Published:** 2015-01-08

**Authors:** Andrei N. Lupas, Hongbo Zhu, Mateusz Korycinski

**Affiliations:** Department of Protein Evolution, Max-Planck-Institute for Developmental Biology, Tuebingen, Germany; UT Southwestern Medical Center at Dallas, United States of America

## Abstract

Despite having caused one of the greatest medical catastrophies of the last century through its teratogenic side-effects, thalidomide continues to be an important agent in the treatment of leprosy and cancer. The protein cereblon, which forms an E3 ubiquitin ligase compex together with damaged DNA-binding protein 1 (DDB1) and cullin 4A, has been recently indentified as a primary target of thalidomide and its C-terminal part as responsible for binding thalidomide within a domain carrying several invariant cysteine and tryptophan residues. This domain, which we name CULT (cereblon domain of unknown activity, binding cellular ligands and thalidomide), is also found in a family of secreted proteins from animals and in a family of bacterial proteins occurring primarily in δ-proteobacteria. Its nearest relatives are yippee, a highly conserved eukaryotic protein of unknown function, and Mis18, a protein involved in the priming of centromeres for recruitment of CENP-A. Searches for distant homologs point to an evolutionary relationship of CULT, yippee, and Mis18 to proteins sharing a common fold, which consists of two four-stranded β-meanders packing at a roughly right angle and coordinating a zinc ion at their apex. A β-hairpin inserted into the first β-meander extends across the bottom of the structure towards the C-terminal edge of the second β-meander, with which it forms a cradle-shaped binding site that is topologically conserved in all members of this fold. We name this the β-tent fold for the striking arrangement of its constituent β-sheets. The fold has internal pseudosymmetry, raising the possibility that it arose by duplication of a subdomain-sized fragment.

## Author Summary

In the public perception, thalidomide mainly evokes children with stunted limbs. Less known is that thalidomide continues to be a very useful drug, licensed in most countries for the treatment of multiple myelomas and leprosy. Aside from its catastrophic effect on human embryonal development, it has a manageable spectrum of side-effects and a broad range of potential indications. Interest in its further pharmacological development thus remains high, but is hindered by our limited knowledge of the reasons for its worst side-effect – teratogenicity. For half a century, even the main protein target of thalidomide in the human body remained unknown, until a seminal study showed in 2010 that this was cereblon. Further progress towards a mechanistic understanding has however been limited by the difficulties in using cereblon for biochemical studies. Here we show that the thalidomide-binding region of cereblon is contained within a domain also present in other protein families, and that this domain is related to several domains with known functions. Where established experimentally, all these domains are seen to form their main substrate-binding sites at the same location in the common fold, often using residues in equivalent positions. Our findings offer the possibility to develop model systems for the study of specific aspects of cereblon activity.

## Introduction

Thalidomide was provided to pregnant women as an anti-nausea and sedative drug from 1957 to 1962, and was available over the counter in many countries. It was withdrawn after it became apparent that it had caused a range of birth defects in many newborns, with over 10,000 cases reported from more than 46 countries [1], [2]. Soon after its ban, however, it was reintroduced as an agent against a complication of leprosy [2]–[4], due to its anti-inflammatory and immunomodulatory activity, and has since then also been evaluated for treatment of, among others, AIDS and Crohn's Disease [5]. In 1994, evidence of its antiangiogenic activity led to its consideration for cancer therapy [6] and it is one of the main drugs available today against multiple myeloma [7], [8]. Despite strict controls on its use, its value in the treatment of leprosy leads to the ongoing birth of babies with thalidomide-induced malformations in developing countries [2], [9].

Because of its antiangiogenic and immunomodulatory activities, pharmacological interest in thalidomide continues to be very high [5], but until recently, its molecular mechanism of action – both with respect to its positive and its negative effects – remained unclear due to a lack of known targets. In 2010, Handa and co-workers showed in a landmark study that cereblon, a protein originally identified in a screen for mutations causing mild mental retardation [10], is a major target of thalidomide and is responsible for the teratogenic effects of the drug [11]. Cereblon owes its name to its involvment in brain development and to its central LON domain, which led to its initial annotation as an ATP-dependent Lon protease [10]. In the 2010 study, Handa and co-workers showed that cereblon is a cofactor of damaged DNA-binding protein 1 (DDB1), which acts as the central component of an E3 ubiquitin ligase complex and regulates the selective degradation of key proteins in DNA repair, replication and transcription [12]. Binding of thalidomide to a C-terminal region in cereblon inhibits the E3 ubiquitin ligase activity of the complex and leads to developmental limb defects in chicks and zebrafish [11]. Point mutations in this region, which abolish thalidomide binding, but allow the continued formation of the E3 complex, restore ubiquitination and prevent the teratogenic activities of thalidomide.

Despite these advances, there has been little progress in understanding the mechanism of thalidomide binding and teratogenicity, due to the difficulties in preparing cereblon protein for biochemical and biophysical studies. Such progress would however be important for further pharmacological development, given that current thalidomide derivatives, such as pomalidomide and lenalidomide, appear to have inherited its teratogenicity (see e.g. [13]). In search of a better understanding of cereblon, we decided to subject the protein to a detailed bioinformatic analysis, with a particular focus on its thalidomide-binding domain. Here we show that this domain is present in several protein families of eukaryotes and bacteria, is related to the highly conserved yippee and Mis18 proteins of eukaryotes, and has a homologous origin with methionine sulfoxide reductase B, the regulatory domain of RIG-I helicase, and glutathione-dependent formaldehyde-activating enzyme. Our findings place the domain into a broad evolutionary context and show that the development of model systems is possible in order to study specific aspects of cereblon activity.

## Results

### The domain structure of cereblon

**Figure 1:**
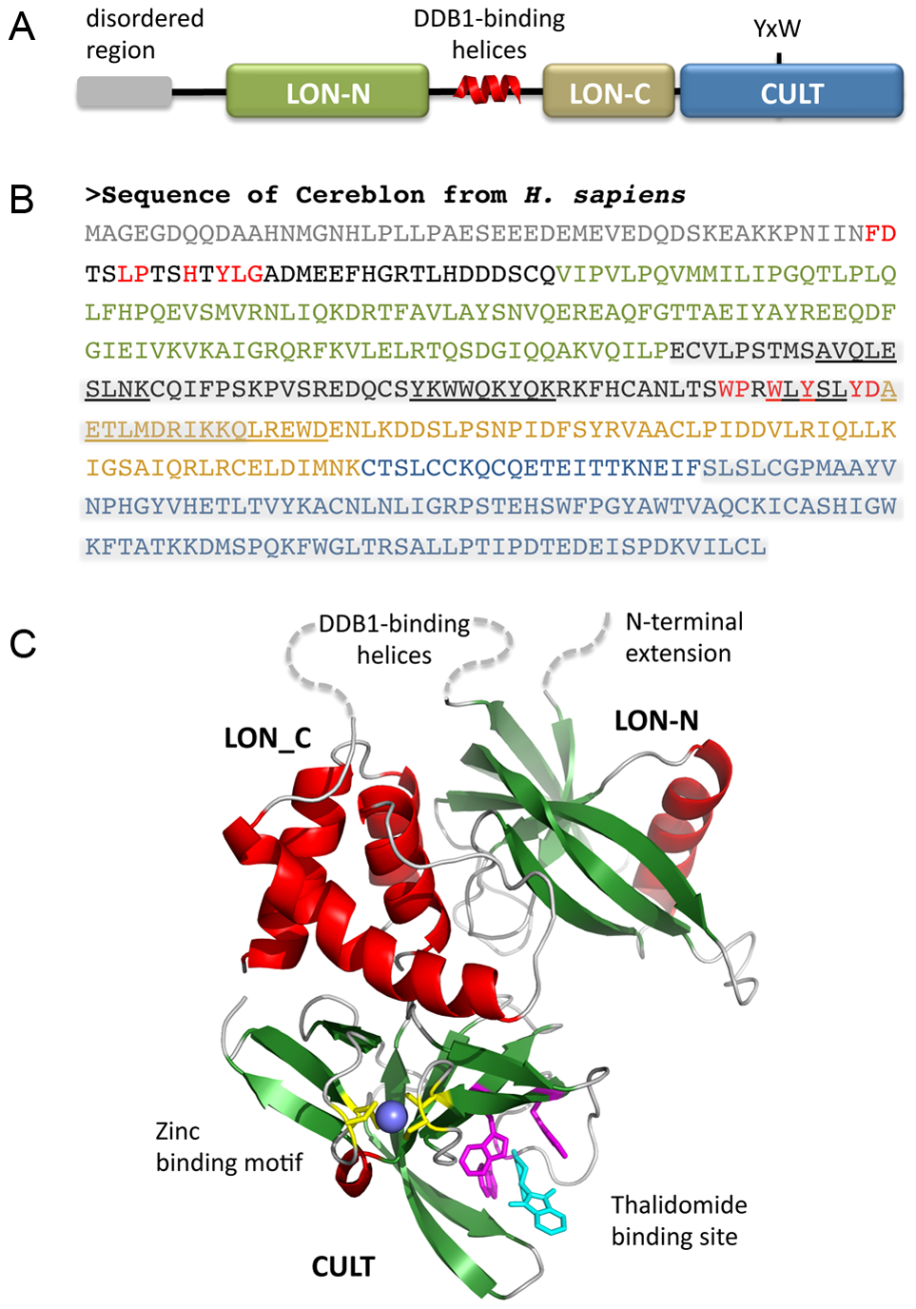
Domain structure of cereblon. (**a**) Schematic view of the domains and motifs in cereblon. The Tyr and Trp residues of the CULT domain whose mutation abolishes thalidomide binding (Ito et al., 2010) are marked. (**b**) Annotated sequence of human cereblon. The two LON domains and the CULT domain are colored as in panel (a). Highly conserved sequence motifs outside these domains are colored red. The two regions identified by deletion analysis as responsible for DDB1 binding (mid-protein) and thalidomide binding (C-terminal) are highlighted in grey. Sequences with high helical propensity in the putative DDB1-binding region are underlined. (**c**) Model of cereblon. The LON domain was modeled on PDB:1ZBO. The N-terminal extension and the large connector between the LON subdomains are marked by dotted lines. The CULT domain was modeled on the structure of a bacterial CULT domain which we have determined [18]. Note that the CULT domain of human cereblon can also be modeled with good accuracy on the homologous structures of MsrB and RIG-I (Fig. S1). After submission of this manuscript, two cereblon-DDB1 complex structures [15], [16] have confirmed the domain arrangement proposed in this model.

Cereblon proteins occur throughout eukaryotes, however not in fungi. They are typically 400–600 residues long (442 in the case of human cereblon) and their genes occur in single copy per genome. Their most salient feature is the presence of a central LON domain (residues 80–317 in human cereblon, Fig. 1). As defined in the Pfam and SMART databases, the LON domain actually comprises two domains, an N-terminal pseudo-barrel of six β-strands, closed off on one side by a helix, (LON-N) and a helical bundle of four to five helices – four in the case of cereblon, to judge by secondary structure prediction and length of the domain (LON-C).

The two LON domains are connected by an unstructured loop of typically around 10 residues, which however is much longer in cereblon, at about 60 residues. Handa and co-workers identified this region by deletion analysis as responsible for DDB1 binding (Fig. 1) [11]. Since an α-helical motif – the H-box – has been found to be a crucial structural element used by both viral and cellular substrate receptors to bind to DDB1 [14], we surmised that an H-box exists in cereblon as well, in the segment connecting LON-N with LON-C. H-box sequences are however very divergent and the H-box is thus primarily defined by its helical propensity and a general pattern of hydrophilic and hydrophobic residues [14]. We tried to identify an H-box in cereblon, and particularly in the connector between the two LON domains, by searching against a profile HMM generated from the H-box sequences listed by Li *et al.* [14], but did not obtain statistically significant matches. The connector is poorly conserved across phyla, but contains a highly conserved motif WPxWxYxxYD immediately prior to the start of the LON-C domain (Fig. 1). Since this motif coincides with a region of elevated helical potential, we considered this the best guess for the site of DDB1 interaction. After submission of this manuscript, two studies presented the structure of cereblon in complex with DDB1 [15], [16], which showed that the interaction between the two proteins is topologically novel and not mediated by an H-box. All three regions with elevated helical propensity in the connector (Fig. 1) are indeed helical, albeit the first one as a 3_10_ helix. The main interactions are formed between the first region and DDB1 β-propeller A, and between the third region (containing the conserved motif) and DDB1 β-propeller C.

N-terminally to the LON domain, cereblon proteins have an extension of typically 50–100 residues (79 in the case of human cereblon), of which the front part is not conserved across phyla and generally predicted as intrinsically unstructured (Fig. 1). Starting about 35 residues prior to the beginning of the LON domain, the extension becomes well-conserved and particularly a motif FDxxLPxxHxYLG is recognizable in most cereblon homologs, from humans and plants to basal eukaryotes. Since the extension is adjacent to the connector between LON-N and LON-C in the LON domain model (Fig. 1) and experimental evidence suggested the binding interactions with DDB1 to be bipartite [14], we considered that this motif could contribute to DDB1 binding. The cereblon-DDB1 complex structures however show that the extension interacts with the C-terminal region of cereblon via the conserved motif [15], [16]. Indeed, since it runs alongside the thalidomide-binding site, it may relay information on the occupancy of the site to the LON-N domain. We therefore conjecture that deletion of the extension could uncouple the C-terminal region from the rest of the E3 ligase complex, alleviating or even entirely abolishing the effects of thalidomide binding.

The C-terminal region of cereblon, comprising about 100–130 residues (125 in the case of human cereblon), represents the best-conserved part. It has multiple invariant residues and encompasses completely the part of the protein identified through deletion analysis as responsible for thalidomide binding [11]. Sequence similarity searches show that this region also occurs in much shorter proteins than cereblon, where it essentially covers the entire length of the protein, identifying it as a domain. We name this domain CULT, for cereblon domain of unknown activity, binding cellular ligands and thalidomide.

### The CULT domain

**Figure 2:**
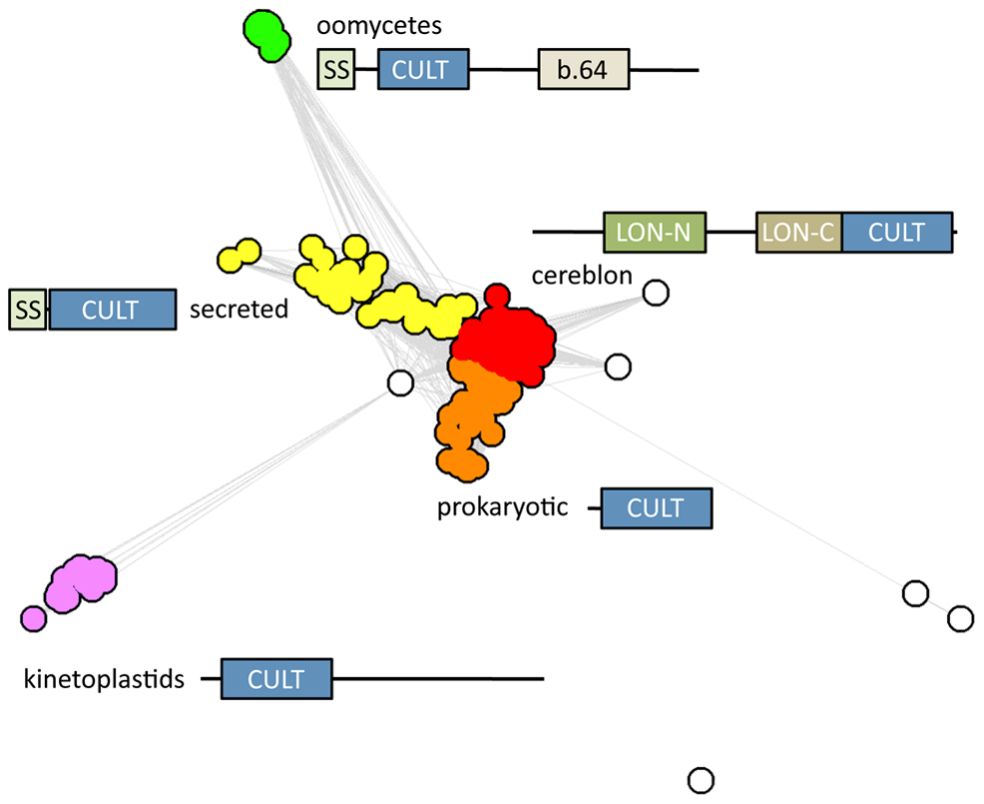
Cluster map of CULT domain proteins. The main clusters are named as described in the text and the domain architecture of the proteins in the respective cluster is shown. For this map, we searched the nr database at NCBI with PSI-Blast, using the CULT domain of human cereblon as a query. After convergence, we extracted all proteins above the cutoff of E = 0.005 and clustered them in CLANS using their all-against-all pairwise similarities as measured by BLAST P-values. Clustering was done to equilibrium in 2D at a P-value cutoff of 1e-10 using default settings.

With the CULT domain of human cereblon as a starting point, PSI-Blast searches of the non-redundant protein database at NCBI converge in three iterations. Analysis of the results, for example using clustering by pairwise sequence similarity in CLANS (Fig. 2), shows that most of the search space consists of cereblon sequences, recognizable by their LON domain, but that several other groups of CULT domain-containing proteins are identifiable. The two main groups are: (I) prokaryotic proteins, mainly from δ-proteobacteria, but with a few representatives from α- and γ-proteobacteria and one sequence from a spirochete; these proteins consist entirely of the CULT domain; and (II) animal proteins from placozoans to vertebrates, but not occurring beyond fishes; these proteins also consist of the CULT domain, but carry an N-terminal secretion signal sequence. Indeed, the homolog from the sand fly *Phlebotomus arabicus* has been identified experimentally as a salivary protein [17]. Two further, more divergent groups are also apparent: one from oomycetes, with an N-terminal signal sequence followed by a CULT domain and ending with a carbohydrate-binding domain (SCOP: b.64); and the second from kinetoplastids, with an N-terminal CULT domain followed by a C-terminal region that cannot be assigned to a known domain family at present. The remaining few sequences from the PSI-Blast search do not recognizably belong to any of these groups; they are mainly from green algae and can all be confirmed by reverse PSI-Blast searches to contain a CULT domain.

**Figure 3:**
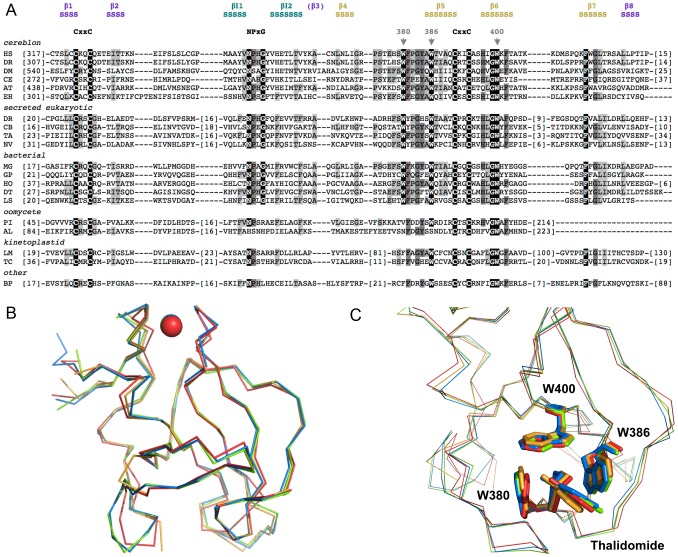
CULT domain sequence and structure. (**a**) Multiple alignment of CULT domains from representative members of the groups in Fig. 2. The alignment is based on the results of the PSI-Blast search with the CULT domain of human cereblon (first sequence in the alignment). Invariant residues of the three core groups (cereblon, secreted eukaryotic, bacterial) are underscored in black, residues conserved in at least two thirds of the sequences in the alignment are highlighted in dark grey and residues in at least one third of the sequences in light grey. The three tryptophan residues forming the thalidomide-binding site are marked by arrowheads and the two cysteine motifs coordinating the Zn ion, as well as a highly conserved motif at the tip of the inserted β-hairpin, are written out. The secondary structure above the alignment (S = β-strand) is the experimentally determined structure of the CULT domain from MGR_0879 of *Magnetospirillum gryphiswaldense* (first bacterial sequence in the alignment; [18]). The β-strands of the two main β-sheets are numbered according to the consensus structure of the β-tent fold and colored by whether they belong to the N-terminal β-sheet (purple) or the C-terminal one (gold); β3 is shown in brackets as it has lost its β-strand character in the CULT domain. The two β-strands of the inserted hairpin (teal) are labeled βI1 and βI2. The sequences are: (cereblon) HS - *Homo sapiens* NP_057386.2, DR - *Danio rerio* NP_001003996.1, DM - *Drosophila mojavensis* XP_001999319.1, CE - *Caenorhabditis elegans* NP_502300.2, AT - *Arabidopsis thaliana* NP_850069.1, EH - *Entamoeba hystolitica* XP_657530.1; (secreted eukaryotic) DR - *Danio rerio* NP_001121712.1, CB - *Caenorhabditis brenneri* EGT59438.1, TA - *Trichoplax adhaerens* XP_002115135.1, NV - *Nasonia virtipennis* XP_003427162.1; (bacterial) MG - *Magnetospirillum gryphiswaldense* CAM74667.1, GP - gamma proteobacterium BDW918 WP_008249149.1, HO - *Haliangium ochraceum* WP_012831591.1, DT - *Desulfonatronospira thiodismutans* WP_008870657.1, LS - *Leptospira* sp. B5-022 WP_020769190.1; (oomycete) PI - *Phytophthora infestans* XP_002999235.1, AL - *Albugo laibachii* CCA16326.1; (kinetoplastid) LM - *Leishmania major* strain Friedlin XP_001681231.1, TC - *Trypanosoma cruzi* EKG02463.1; (other) BP - *Bathycoccus prasinos* XP_007508760.1. (**b**) Superimposition of bacterial and eukaryotic CULT domain structures from *M. gryphiswaldense* (red), *H. sapiens* (blue; PDB ID:4TZ4), *M. musculus* (orange; PDB ID: 4TZC), and *G. gallus* (green; PDB ID:4CI2). The r.m.s. deviations in Cα positions for the pairwise comparisons ranged between 0.4 and 0.9 Å and are listed in S1 Table. (**c**) Superimposition of the thalidomide binding site for the structures in panel (b). The ligand and the residues of the aromatic cage are shown in stick representation and colored as in panel (b). Residue numbering is for the human protein.

Multiple alignment of the sequences identified in the search (Fig. 3A) show that several residues are highly conserved in the CULT domain. Conservation of these residues is highest in the CULT core group (cereblon, secreted eukaryotic sequences, bacterial sequences) and declines towards the periphery. Particularly conspicuous are three tryptophan residues, marked by arrows in Fig. 3A, which are seen to form the binding site for thalidomide and cellular ligands in the crystal structure of the bacterial CULT protein MGR_0879 from *Magnetospirillum gryphiswaldense* ([18]; PDB ID:4V2Y; Figs. 3B,C). The crystal structure, which we determined after this bioinformatic study, shows that the other highly conserved residues group around this binding site (S1 Fig.), their conservation being rationalized by an influence on substrate recognition and discrimination. The one exception to this are two CxxC cysteine motifs, which we took from the beginning of this project to be indicative of a zinc binding site and thus present for structural reasons.

We developed the bacterial model system to study the CULT domain because we found eukaryotic cereblon proteins very difficult to express in useful amounts and even more difficult to purify in a soluble state. In contrast, the bacterial protein could be produced and purified in a straight-forward way [18]. We reasoned that, at 36% sequence identity and with almost all well-conserved positions similar or the same between the CULT domains of humans and *Magnetospirillum*, the bacterial system should represent an accurate model for the eukaryotic domain. The structures of eukaryotic CULT domains from human, mouse and chicken [15], [16] now show that the expectation is true to an astonishing extent, with a root-mean-square deviation (r.m.s.d.) of around 0.9 Å over 100 Cα positions between the bacterial and human proteins (Fig. 3B). The bacterial domain is thus structurally almost as similar to the eukaryotic domains as these are to each other (Table. S1).

### The β-tent fold

**Figure 4:**
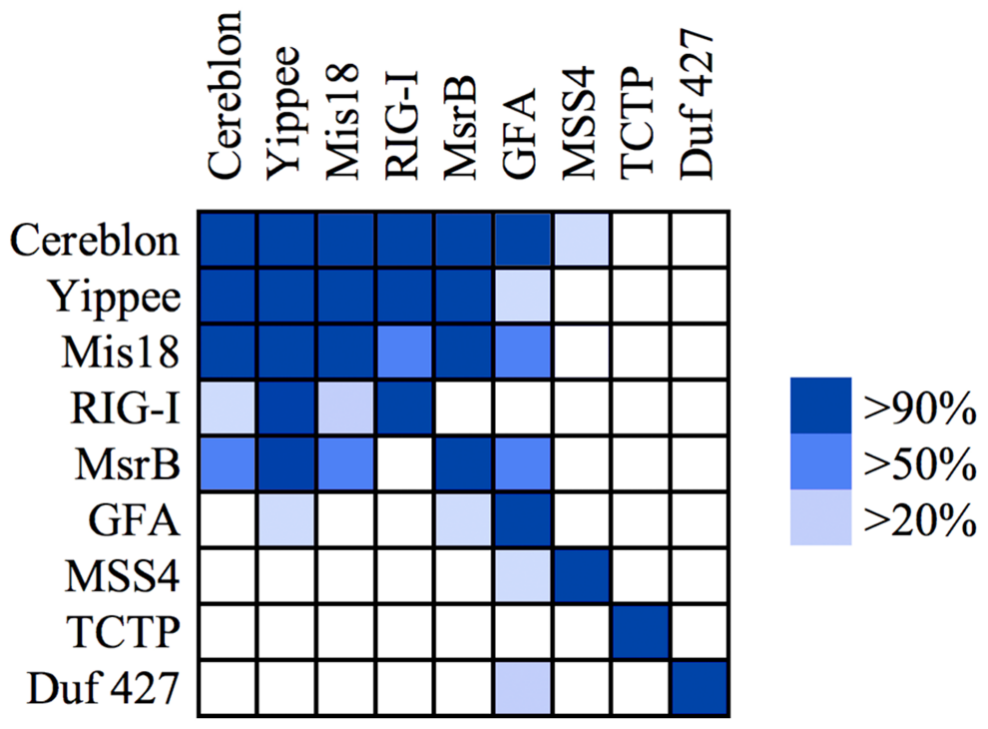
Sequence relationships between proteins of the β-tent fold. The map shows the probabilities obtained for HMM to HMM comparisons, as implemented in HHpred. Queries are in rows, targets in columns. The proteins are: cereblon - CULT domain of human cereblon; Yippee - *Drosophila melanogaster* yippee isoform A (AAF48266.1); Mis18 - *Schizosaccharomyces pombe* Mis18 (CAB72327.2), res. 1–125; RIG-I - PDB: 4A2V; MsrB - PDB: 3HCG; GFA - PDB: 1X6M; MSS4 - PDB: 2FU5; TCTP - PDB: 1YZ1; Duf 427 - PDB: 3DJM.

**Figure 5:**
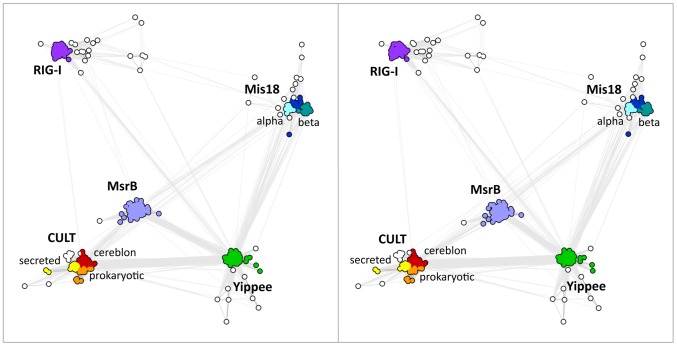
Cluster map of CULT domain homologs. The image is a cross-eyed stereo view and shows that the five domain families occupy the vertices of an approximately equilateral pyramid. For this map, we searched the nr database at NCBI with PSI-Blast, using the CULT domain protein MGR_0879 of M. gryphiswaldense as a query. After six iterations, we extracted all proteins above the cutoff of E = 0.005 and clustered them in CLANS using their all-against-all pairwise similarities as measured by BLAST P-values. Clustering was done to equilibrium in 3D at a P-value cutoff of 1e-5 using default settings.

We searched for remote homologs of the CULT domain using profile Hidden Markov Model (HMM) comparisons in HHpred and obtained matches at probabilities better than 90% (E values <1e-6) for multiple protein families, several of which have members of known structure (Fig. 4). The best matches were to a protein family found throughout eukaryotes, yippee [19]. Two of the five yippee paralogs in mammals have been implicated in signal transduction [20] and tumor suppression [21], respectively, but the actual mechanism of these proteins remains unknown. In searching against Pfam we noticed that, in this database, the profile for yippee (PF03226) was generated jointly with another protein, Mis18, which in our analyses is not particularly close to yippee and indeed seems about as remote from yippee as cereblon is (Fig. 5). Mis18 proteins are broadly represented in eukaryotes, except plants, and appear to be involved in centromere assembly [22], [23], although their actual mechanism remains unknown. The reason for merging Mis18 with yippee in Pfam is unclear to us.

Two protein families of known structure related at a similar level to the CULT domain as yippee and Mis18 are methionine sulfoxide reductase B (MsrB or SelR) and the regulatory domain of retinoic acid-induced gene-1 (RIG-I). MsrB is the most widely distributed protein in this study and is universal to all cellular life. It protects cells from oxidative stress by reducing methionine-R-sulfoxide residues (for a review see e.g. [24]). RIG-I has a more limited phylogenetic spectrum, being detectable only in animals. It is an RNA helicase that, upon binding viral RNA, activates the host innate immune system (for a review see e.g. [25]). The regulatory domain is the RNA 5′-triphosphate sensor of RIG-I, activating the ATPase activity of the protein by RNA-dependent dimerization [26]. These four proteins, yippee, Mis18, MsrB, and RIG-I, are sufficiently close to the CULT domain in sequence space that they usually show up in the non-significant part of sequence similarity searches, between E values of 0.005 and 10, and are occasionally included in the significant part as well. Thus, for example, PSI-Blast searches of the nr database with our bacterial model protein, *Magnetospirillum* MGR_0879, include the first yippee and RIG-I sequences in the second iteration and the first Mis18 and MsrB sequences in the fifth. These proteins appear roughly equidistant from cereblon in sequence space (Fig. 5). More distantly related, but showing up with fair regularity in our searches is glutathione-dependent formaldehyde-activating enzyme (GFA), a protein found in bacteria and most eukaryotes, except plants. GFA catalyzes the first step in the detoxification of formaldehyde [27].

**Figure 6:**
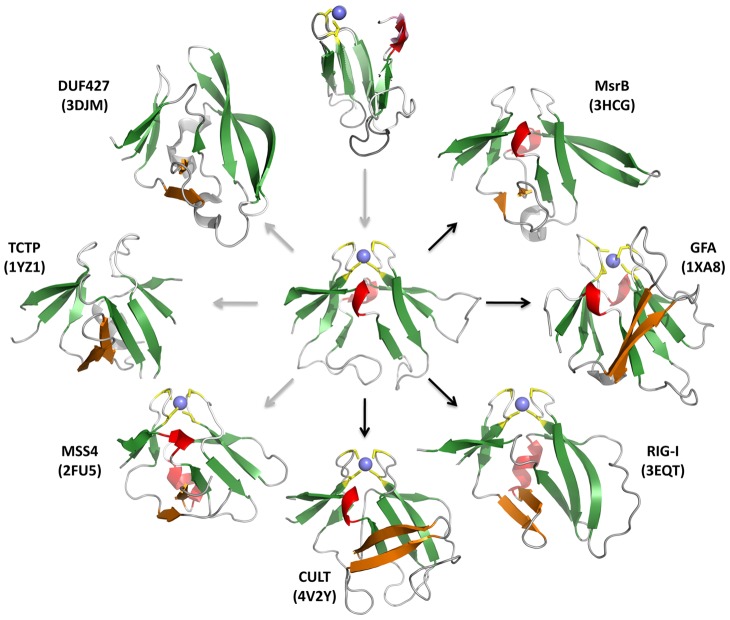
Structure gallery and evolutionary inference for proteins with a β-tent fold. Only the core fold is shown; in structures where additional parts of the polypeptide chain obscured the view on the core fold, these were omitted. The β-strands of the insertion between β2 and β3 of the N-terminal β-meander is colored orange. The top image shows a superposition of the two halves of the MsrB structure 3HCJ (r.m.s.d. of 1 Å over the Cα carbons of the 30 superimposable residues) and the central image shows 3HCJ itself, as the most symmetrical of the β-tent structures. The images around the circumference show the seven domains of known structure discussed in this article (see also Fig. S2). Of these, DUF427 and TCTP systematically lack a zinc binding site, and MsrB homologs have occasionally lost it. In the other domains, the zinc binding site is essentially always present, although the cysteine pattern is slightly modified in GFA relative to all other domains, the first cysteine tandem being CxCxxx, rather than xxCxxC. The arrows in the figure show our inference for a possible evolutionary path. The fold could thus have originated by duplication of a four-stranded β-meander and subsequently diverged into the domains seen today. Where homologous relationships are supported by sequence similarity, the arrows are black; otherwise they are grey.

All these proteins share a common fold, formed by two four-stranded, antiparallel β-sheets that are oriented at approximately a right angle and pinned together at their tip by a zinc ion (Fig. 6). The two sheets are connected covalently across the top on both sides by loops, due to circular permutation. Thus, the last strand of the domain is topologically the first strand of the first sheet, yielding the strand order β8-β1-β2-(β3) for the first sheet and (β4)-β5-β6-β7 for the second (β3 and β4 are shown in brackets as, in some structures, they have lost their β-strand character). Because of the striking arrangement of these β-sheets we have named this fold the β-tent.

**Figure 7:**
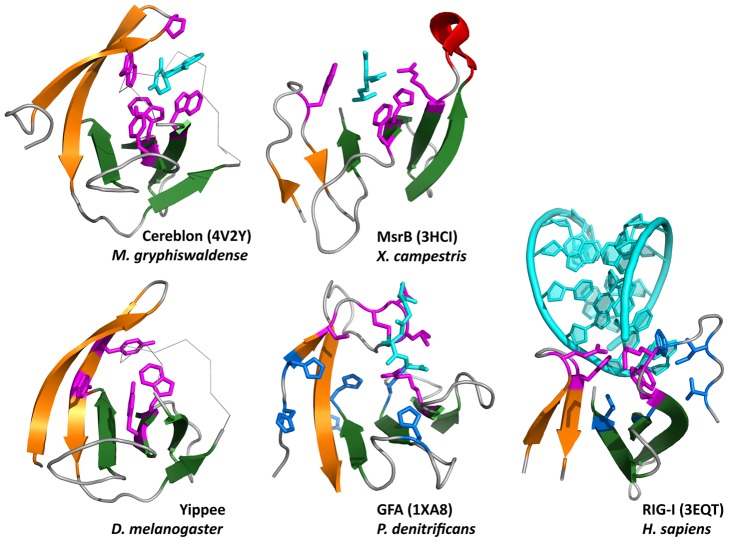
Gallery of β-tent domain binding sites. The panels show the β-hairpin inserted between β2 and β3, and the C-terminal β-meander (β4–β7), colored as in Fig. 6. Residues involved in ligand binding are colored magenta and blue, and the ligands cyan. Yippee is the molecular model from Fig. S1 and the ligand-binding residues are predicted based on conservation and location in the fold. For GFA, only the residues involved in binding the glutatione cofactor are known (magenta). Six highly conserved histidine residues (blue) may participate in catalysis, but the exact location and geometry of the formaldehyde-binding site is unknown. For Rig-I, the residues involved in coordinating the RNA 3′ end are shown in magenta and the 5′ end in blue. Residue numbers are: Cereblon: P51, W79, W85, W99, Y101, Yippee: Y43, F45, W82, Y84, MsrB: W73, R97, H111, F113, GFA: C54, T57, L58, C56, R98 (magenta) and H32, H50, H52, H107, H117, H126 (blue), RIG-I: E573, H576, W604, K602 (magenta) and V632, L621, V595, I597, F601, W604 (blue).

A conserved feature of all proteins with a β-tent fold is an insertion between strands β2 and β3, which usually has a β-hairpin stem and reaches across the bottom of the tent to extend the second β-sheet at its C-terminal edge. Due to the curvature of the β-sheet and the sizable nature of the loops connecting β4 to β5 on one side and the strands of the insertion on the other, all β-tent proteins contain a cradle-shaped groove at this location, which hosts the binding site (Fig. 7).

**Figure 8:**
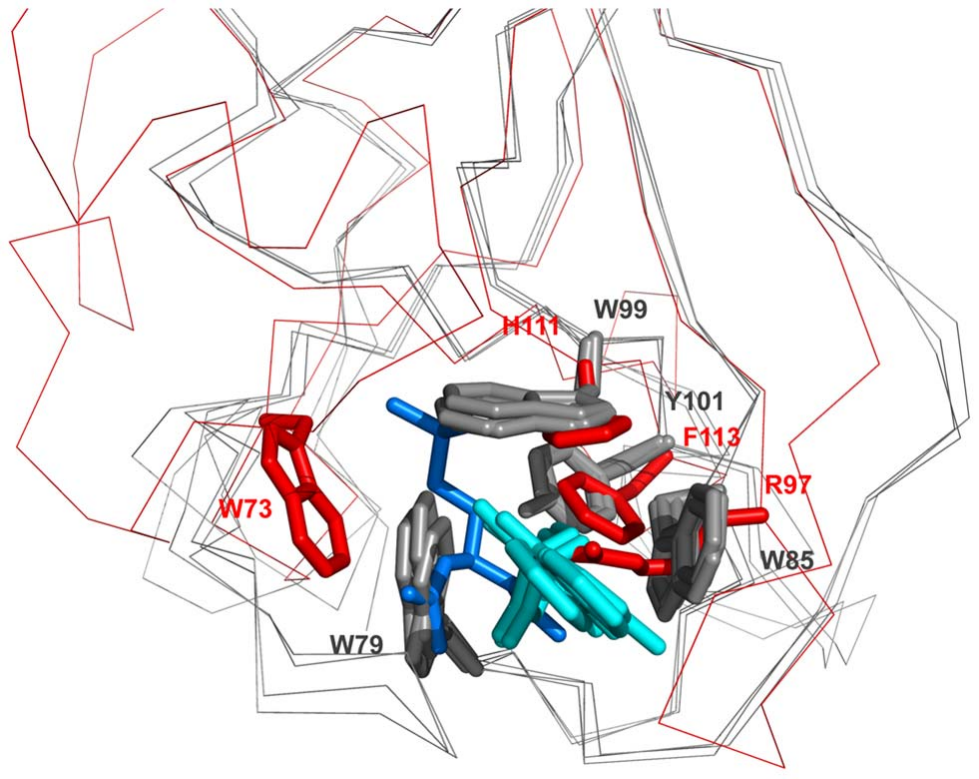
Comparison of the substrate-binding sites in CULT and MsrB. The superimposition shows the thalidomide binding sites in the CULT domain structures (*M. gryphiswaldense* MGR_0879, *G. gallus* 4CI2, *M. musculus* 4TZC) in gray and the methionine sulfoxide binding site of MsrB (*X. campestris* 3HCI) in red. The ligands are colored cyan (thalidomide) and marine blue ((2S)-2-(acetylamino)-N-methyl-4-[(R)-methylsulfinyl]butanamide). Residue numbers are provided for the *M. gryphiswaldense* structure in gray, and for MsrB in red.

The residues giving the binding site its specificity in the individual proteins are frequently found in equivalent positions. This is particularly conspicuous when comparing the binding sites of CULT and MsrB (Fig. 8). Of the four residues forming the thalidomide-binding site in *Magnetospirillum* CULT (4V2Y: W79, W85, W99, and Y101), the last three have equivalents in homologous positions in the methionine sulfoxide-binding site of MsrB (3HCI: R97, H111, F113); the first, W79, is also a tryptophan in the MsrB binding site, but from an analogous position in the insert loop (W73), due to a shift in the position of the site caused by the shape difference between W85 of CULT and R97 of MsrB. This shift places the ligand above β7 in MsrB, rather than above β6, allowing the positioning of a further residue into the active site, which is the catalytic cysteine; conversely, there appears to be no need for a catalytic residue in CULT. We note that the homology of the conserved aromatic residues in CULT to the residues of the binding site in MsrB can be readily seen from the HHpred alignment.

Extending these observations to yippee, which has a similar distribution of conserved residues as CULT (S1 Fig.), we predict that the binding site of this protein is also an aromatic cage, comprising the highly conserved Y43, F45, W82, and Y84, as numbered in *D. melanogaster* yippee isoform B, ABC67182.1 (Fig. 7). Of these, W82 and Y84 are in homologous positions to W99 and Y101 of CULT and H111 and F113 of MsrB, whereas Y43 and F45 are at the same position as W73 in MsrB, but not recognizably homologous.

A striking property of the β-tent fold is that, in several of the proteins, the two sheets have considerable structural symmetry, such as for example in the MsrB structure 3HCJ, where superposition of the two 43-residue halves yields an r.m.s.d. of 1 Å over the Cα positions of the core 30 residues (Fig. 6). This raises the possibility that the fold originated by duplication of a subdomain-sized fragment, but we note that no similarity is detectable between the two halves by sequence comparisons.

Searches in structure space for other proteins with the β-tent fold yielded three more proteins of known structure (Figs. 6, S2), which share the fold with the same topology of secondary structure elements, including the β-hairpin extension between strands β2 and β3, but have no significant sequence similarity to the other proteins in this study, or to each other (Fig. 4). These are MSS4, a guanine exchange factor and nucleotide-free chaperone for the Rab GTPase [28], [29], TCTP, a pleiotropic protein involved in malignant transformation and regulation of apoptosis [30]–[32], and DUF427, a domain of unknown function. Whereas MSS4 and TCTP are eukaryotic proteins, TCTP being present universally and MSS4 broadly, but not in plants, DUF427 is seen mainly in bacteria and fungi, with a small number of archaea presumably having acquired this domain by lateral transfer. Of these proteins, only MSS4 has the zinc binding site (Fig. 6). In the SCOP database, MSS4 and TCTP are grouped together with MsrB and GFA as families within the MSS4-like superfamily, which is the sole representative of the MSS4-like fold (b.88).

### What are the physiological ligands of CULT?

**Figure 9:**
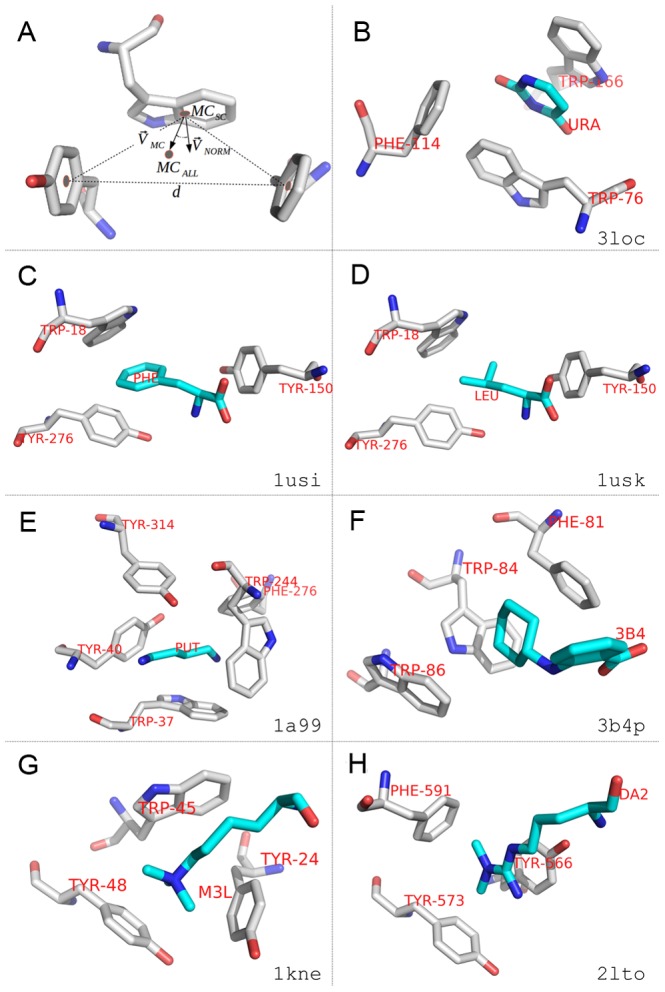
Structural gallery of aromatic cage-like conformations and associated ligands. Aromatic residues forming cage-like conformations are depicted in gray, ligands in cyan. Oxygen: red; nitrogen: blue; sulphur: yellow. Panels **b–h** show examples from the five main categories (S2 Table); their PDB accession codes are shown at the bottom-right corner of each panel. Cage residues are labeled in red by their three-letter names and residue numbers from the PDB entry. Ligands are marked by their three-letter identifiers taken from PDB. (**a**) Geometric criteria for detecting aromatic cage-like conformations (see Method section for a detailed description). (**b**) Heterocyclic ring (uracil) bound to the pyrimidine-sensing transcriptional regulator RutR. (**c**) Hydrocarbon ring (tyrosine) bound to the periplasmic leucine-binding protein of *E. coli*. (**d**) Hydrocarbon chain (leucine) bound to the same binding site (1a99). (**e**) Hydrocarbon chain containing N/O/S (putrescine) bound to the periplasmic putrescine-binding protein of *E. coli*. (**f**) Hydrocarbon ring (2-(cyclohexylamino)benzoic acid) bound to the phenazine biosynthesis protein PhzA/B from Burkholderia cepacia R18194. (**g**) Ammonium cation (metyllysine 9 of histone H3) bound to the *Drosophila* HP1 chromodomain. (**h**) Ammonium cation (dimethylarginine) bound to the Tudor domain of human TDRD3.

A fundamental issue in understanding the biological role of CULT domains, not directly illuminated by their homology to other proteins, is the identification of their physiological ligand(s). The only ligands known today, thalidomide and its derivatives, are clearly non-physiological. Given that the clustering of the invariant tryptophans into a cage-like arrangement was already suggested at the modeling stage (see above), we searched PDB for ligands bound in aromatic cages, loosely defined. For this we allowed the aromatic residues to be Phe and Tyr, as well as Trp, and provided only a very general requirement for cage-like geometry, in order to gain as broad a view as possible (see Methods). We obtained 1098 distinct ligands, which could be grouped approximately into five classes, corresponding to heterocyclic rings, hydrocarbon rings, hydrocarbon chains with and without heteroatoms, and ammonium-based cations (Fig. 9, Table S2). Upon inspection, many of the “cages” identified indeed turned out to be only approximately cage-like and for 46 ligands, all binding sites turned out to be geometrically too divergent to be considered further.

Half of the identified ligands belonged to the largest class, comprising heterocyclic rings. Many of these were enzyme inhibitors, both of natural and synthetic origin, such as indole-2,3-diones (4KWG), aryl hydrazines (4MQQ), or non-nucleoside reverse transcriptase inhibitors (1S9G). Thalidomide, which is bound in the aromatic cage of CULT domains via its glutarimide ring, belongs to this class. Among the natural compounds, we found pyrimidines and their nucleosides of particular interest, as these resemble the glutarimide ring of thalidomide [18] and are bound in similar cages. For example, the transcription factor RutR (3LOC; Fig. 9B) can bind both uracil and thymine in its aromatic cage and acts as the master regulator of genes involved in the synthesis and degradation of pyrimidines [33]. An experimental screen against *Magnetospirillum* MGR_0879 found that, of the nucleobases, uracil and its nucleoside (uridine) were indeed bound and their relevance for eukaryotic cereblon could be established *in vivo* in zebrafish. It is attractive to consider that they might also be the physiologically relevant ligand, given that DDB1 is an integrator of cellular information on DNA damage and incorporation of uracil into DNA represents a mutagenic lesion [18].

Another type of ligand that we found to be of particular interest in this analysis comprises amino acid sidechains, modified and unmodified. These include the heterocyclic rings of His, Pro, and Trp, the hydrocarbon rings of Phe (Fig. 9C) and Tyr, the hydrocarbon chains of Ile, Leu (Fig. 9D), Met, and Val, and the cationic sidechains of metylated and unmethylated Lys (Fig. 9G) and Arg (Figs. 9H, S3). Particularly the latter occur prominently in the tails of histones and are recognized by aromatic cages in a range of different domains, including bromodomains, chromodomains (Fig. 9G), and Tudor domains (Fig. 9H). Given that the DDB1-Cul4A E3 ubiquitin ligase complex is known to bind and ubiquitinate histones (see e.g. [34]), an activity of cereblon in recognizing histone tail modifications within a linear sequence motif and providing the target specificity for the ligase complex appears fully plausible [18].

Other sidechain interactions, particularly in the context of linear sequence motifs, also appear entirely possible. Thus, the homeobox transcription factor MEIS2, which is implicated in various aspects of human development, was recently identified as a cereblon interactor [15]. Its binding was exclusive with thalidomide and its derivatives, suggesting that it is recognized via the same binding site. We note that MEIS2 and its paralogs contain two folded domains, one being the homeobox domain and the other uncharacterized at present, flanked by extended regions predicted to be unstructured and with low sequence conservation. The N-terminal approximately 10 residues are however very highly conserved and contain sidechains (Arg, Tyr, His) that could easily be envisaged as the ligands of an aromatic cage. We therefore consider this region to be the most attractive first candidate for exploring the MEIS2-cereblon interaction. This said, the very high similarity between the bacterial and eukaryotic CULT domains, particularly in the area of the aromatic cage, points to a wide-spread ligand, present also outside the cell, rather than to a linear sequence motif. By similarity to metyllysine, one might envisage choline, carnitine, betaine, and related compounds, but none of these could so far be seen to interact with the CULT domain in our model system.

## Discussion

In this article we have presented evidence that the thalidomide-binding region of cereblon is a conserved domain, CULT, present in several other proteins of eukaryotes and bacteria. The CULT domain is recognizably homologous to at least five other domain families, which share - where known - a common fold and a shared mechanism of ligand binding. The fold is also recognizable in three further domain families, which however do not have detectable sequence similarity to any of the other proteins, or to each other, and whose evolutionary relationship thus remains unclear (the SCOP database, however, clearly considers them homologous, as it groups them into the same superfamily). We have named the common fold of these proteins the β-tent, due to the orientation of its two constituent β-sheets.

The widely differing activities of proteins with a β-tent fold, as well as the absence of invariant residues across the domains, suggest that the β-tent is a structural scaffold, which mounts a binding site at a specific location. The binding site is formed by a cradle-shaped groove, whose sides are provided by loops connecting strands β4 to β5, and βI1 to βI2 of the common fold; the bottom is formed by strands β5, β6, and β7. The elaboration of this site in the individual families is tailored to their specific function, but appears to follow common principles, particularly in families binding small-molecule ligands. Here, binding residues are mainly located on the two loops and strand β6, while catalytic residues appear to be located on strand β7. For families whose binding site is at present unknown, this can therefore be reasonably predicted by mapping their conservation pattern onto homology models of the relevant region.

Members of the β-tent fold show, to varying degrees, a twofold rotational symmetry around a central axis passing through the apical zinc ion (where present). The symmetry is most pronounced in MsrB and this domain also has the broadest phylogenetic spectrum, being the only one with a universal representation in all cellular life forms. It therefore seems attractive to surmise that it is the ancestral representative of this fold, from which the others evolved by duplication and differentiation, and that it itself originated by duplication of a four-stranded β-meander. We have previously argued for an origin of folded proteins from subdomain-sized peptides [35], [36]. But for the apparent lack of internal sequence symmetry to support this inference, the β-tent would seem an attractive candidate for such a scenario.

The absence of statistically significant sequence similarity between MSS4, TCTP, DUF427 and the other proteins of this fold raises the possibility of a convergent origin. We note however that MsrB and RIG-I also do not share statistically significant sequence similarity between each other (Fig. 4) and are only connected conclusively in sequence space via CULT and yippee (Fig. 5). The homology of all proteins with a β-tent fold thus remains a clear possibility, which may become substantiated by new domain families found in hitherto poorly explored parts of the tree of life.

## Methods

Sequence similarity searches were carried out at the National Institute for Biotechnology Information (NCBI; http://blast.ncbi.nlm.nih.gov/) and in the MPI Bioinformatics Toolkit (http://toolkit.tuebingen.mpg.de; [37]). PSI-Blast [38] at NCBI was run on the non-redundant protein sequence database (nr) with an E-value threshold of 0.005. CS-Blast [39] in the MPI Toolkit was run on a version of nr clustered at 70% sequence identity (nr70), also with a threshold of E = 0.005. The sequence relationships of proteins identified in these searches were explored by clustering them according their pairwise Blast P-values [40] in CLANS [41]. Clustering was done in default settings (attract = 10, repulse = 5, exponents = 1), with other settings as given in the figure legends.

Searches for more distant homologs were made with HHpred [42] and HHsenser [43] on the databases pdb70 (sequences of protein databank structures, as available in April 2014, clustered at 70% sequence identity), CDD (conserved domain database from NCBI, as of February 2014), pfamA release 27.0, SCOP release 1.75, and profile HMM databases of all human and all Drosophila proteins built locally and available through the MPI Toolkit.

Secondary structure was predicted in the MPI Toolkit, using the meta-tool Quick2D.

Structure similarity searches were carried out on the Dali server (ekhidna.biocenter.helsinki.fi/dali_server; [44]). Molecular models were built using Modeller [45], and manipulated in Swiss PDB Viewer [46] and PyMol (Schrödinger, LLC). Sequence conservation patterns were visualized with ProtSkin (http://www.mcgnmr.mcgill.ca/ProtSkin/; [47]).

Aromatic cage-like conformations containing ligands in PDB structures were detected by applying a set of geometric criteria (see Figure 9A). First, in each PDB structure, all non-water molecules in the HETATM record were identified and regarded as ligands. Only aromatic residues (phenylalaline, tryptophan and tyrosine) within 6.0 Å distance to these ligands were considered in further analysis. Then, we defined a set of at least three aromatic residues from the same polypeptide chain to form a cage-like conformation interacting with a ligand if: a) all pairwise distances between their side chain mass centers (*MC_SC_*) were less than 10.0 Å; b) the angle between 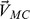 and 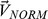 was less than 60° for at least three of the aromatic residues, where 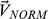 is the normal vector of the aromatic ring, 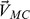 is the vector connecting *MC_SC_* and the mass center of all side chain heavy atoms (*MC_ALL_*); and c) at least two ligand atoms were within 3.0 Å distance to *MC_ALL_*. The program was implemented in Python using *BioPython* [48], *SciPy* [49] and *NetworkX* [50] libraries.

We applied these geometric rules to scan 102,886 PDB files downloaded from the PDB (24 Aug 2014). In total, 6,144 putative aromatic cage-like conformations were detected with 1,098 different ligands binding to them. We grouped the cages according to the ligands they interact with. In each group, redundant cage-like conformations were removed (two cage-like conformations were considered identical if the composite residue names and numbers were the same). Subsequently, we manually examined at least one cage-like conformation in each of the 1,098 groups. Based on the ligand moiety within the aromatic cage, we further classified the 1,098 groups into different categories (S2 Table).

## Supporting Information

S1 Fig **Comparative models of the β-tent domains in (a) human cereblon and (b) Drosophila yippee.** Models are colored according to the templates used (black - CULT domain of M. gryphiswaldense MGR_0879, 4V2Y; red - X. campestris MsrB, 3HCJ; green - human RIG-I, 3EQT). The two central β-hairpins that mount the zinc binding site are shown bold. For each protein, the two following panels show the sequence conservation mapped onto the surface of the model built on the CULT domain of MGR_0879. The red-to-white scale follows highest-to-lowest conservation. Mapping was done using ProtSkin and a multiple sequence alignment derived from two iterations of PSI-Blast and filtered to 70% identity.
(DOC)

S2 Fig **Structure gallery of proteins of the β-tent fold superimposed on the crystal structure of M. gryphiswaldense MGR_0879 (grey).** The superimposition mainly covers the two four-stranded β-sheets; the β-hairpin insertion that reaches across the bottom of the fold to the C-terminal edge of the second β-sheet is present in all folds, but too struturally variable to superimpose well. A superimposition of the best-conserved structural core of all structures in the figure is shown in the center. The r.m.s.deviations in Cα carbons for the pairwise superimpositions were, in clockwise order: 3HCG: 0.98 Å over 63 residues, 1XA8: 1.72 Å over 30 residues, 3EQT: 1.38 Å over 63 residues, 2FU5: 1.40 Å over 57 resiudes, 1YZ1: 1.31 Å over 50 residues, 3DJM: 1.83 Å over 27 residues.
(DOC)

S3 Fig **Protein-protein association mediated by an aromatic cage.** The image shows the main specific contact in the interaction between neuropilin-1 (cyan) and vascular endothelial growth factor-A (VEGF-A) (green), mediated by the docking of the C-terminal arginine of VEGF-A into an aromatic cage formed by neuropilin-1 (PDB: 4DEQ; Parker MW, Xu P, Li X, Vander Kooi CW. Structural basis for selective vascular endothelial growth factor-A (VEGF-A) binding to neuropilin-1 (2012) J Biol Chem 287: 11082–11089).
(DOC)

S1 Table **Structural similarity of the CULT domains from Magnetospirillum MGR_0879 and human, mouse and chicken cereblon.** The numbers show the r.m.s. deviation in Å over the number of superimposed Cα carbons.
(DOC)

S2 Table **Classes of ligands detected in cage-like binding sites in PDB (see also Methods and Fig. 9).**
(DOC)

## References

[1] Lenz W (1988) A short history of thalidomide embryopathy. Teratology 38: 203–215. 3067415 10.1002/tera.1420380303

[2] World Health Organization (2014) Use of thalidomide in leprosy. Available: http://www.who.int/lep/research/thalidomide/en/. Accessed 9 May 2014.

[3] Sheskin J (1965) Thalidomide in the treatment of lepra reactions. Clin Pharmacol Ther 6: 303–306. 14296027 10.1002/cpt196563303

[4] Teo S, Resztak KE, Scheffler MA, Kook KA, Zeldis JB, et al (2002) Thalidomide in the treatment of leprosy. Microbes Infect 4: 1193–1202. 12361920 10.1016/s1286-4579(02)01645-3

[5] Franks ME, Macpherson GR, Figg WD (2004) Thalidomide. Lancet 363: 1802–1811. 15172781 10.1016/S0140-6736(04)16308-3

[6] D'Amato RJ, Loughnan MS, Flynn E, Folkman J (1994) Thalidomide is an inhibitor of angiogenesis. Proc Natl Acad Sci U S A 91: 4082–4085. 7513432 10.1073/pnas.91.9.4082 PMC43727

[7] Singhal S, Mehta J, Desikan R, Ayers D, Roberson P, et al (1999) Antitumor activity of thalidomide in refractory multiple myeloma. N Engl J Med 341: 1565–1571. 10564685 10.1056/NEJM199911183412102

[8] Morgan GJ, Davies FE (2013) Role of thalidomide in the treatment of patients with multiple myeloma. Crit Rev Oncol Hematol 88 Suppl 1: S14–22. 23827438 10.1016/j.critrevonc.2013.05.012

[9] Crawford A (2013) Brazil's new generation of Thalidomide babies. BBC News Magazine Available: http://www.bbc.com/news/magazine-23418102. Accessed 9 May 2014.

[10] Higgins JJ, Pucilowska J, Lombardi RQ, Rooney JP (2004) A mutation in a novel ATP-dependent Lon protease gene in a kindred with mild mental retardation. Neurology 63: 1927–1931. 15557513 10.1212/01.wnl.0000146196.01316.a2 PMC1201536

[11] Ito T, Ando H, Suzuki T, Ogura T, Hotta K, et al (2010) Identification of a primary target of thalidomide teratogenicity. Science 327: 1345–1350. 20223979 10.1126/science.1177319

[12] Iovine B, Iannella ML, Bevilacqua MA (2011) Damage-specific DNA binding protein 1 (DDB1): a protein with a wide range of functions. Int J Biochem Cell Biol 43: 1664–1667. 21959250 10.1016/j.biocel.2011.09.001

[13] D'Amato RJ, Lentzsch S, Rogers MS (2013) Pomalidomide is strongly antiangiogenic and teratogenic in relevant animal models. Proc Natl Acad Sci U S A 110: E4818. 24302770 10.1073/pnas.1315875110 PMC3864340

[14] Li T, Robert EI, van Breugel PC, Strubin M, Zheng N (2009) A promiscuous alpha-helical motif anchors viral hijackers and substrate receptors to the CUL4-DDB1 ubiquitin ligase machinery. Nat Struct Mol Biol 17: 105–111. 19966799 10.1038/nsmb.1719 PMC2823288

[15] Fischer ES, Böhm K, Lydeard JR, Yang H, Stadler MB, et al (2014) Structure of the DDB1-CRBN E3 ubiquitin ligase in complex with thalidomide. Nature 512: 49–53. 25043012 10.1038/nature13527 PMC4423819

[16] Chamberlain PP, Lopez-Girona A, Miller K, Carmel G, Pagarigan B, et al (2014) Structure of the human Cereblon-DDB1-lenalidomide complex reveals basis for responsiveness to thalidomide analogs. Nat Struct Mol Biol 21: 803–809. 25108355 10.1038/nsmb.2874

[17] Hostomská J, Volfová V, Mu J, Garfield M, Rohousová I, et al (2009) Analysis of salivary transcripts and antigens of the sand fly *Phlebotomus arabicus* . BMC Genomics 10: 282. 19555500 10.1186/1471-2164-10-282 PMC2714351

[18] Hartmann MD, Boichenko I, Coles M, Zanini F, Lupas AN, et al (2014) Thalidomide mimics uridine binding to an aromatic cage in cereblon. J Struct Biol 188: 225–232. 25448889 10.1016/j.jsb.2014.10.010

[19] Roxström-Lindquist K, Faye I (2001) The Drosophila gene Yippee reveals a novel family of putative zinc binding proteins highly conserved among eukaryotes. Insect Mol Biol 10: 77–86. 11240639 10.1046/j.1365-2583.2001.00239.x

[20] Liang P, Wan Y, Yan Y, Wang Y, Luo N, et al (2010) MVP interacts with YPEL4 and inhibits YPEL4-mediated activities of the ERK signal pathway. Biochem Cell Biol 88: 445–450. 20555386 10.1139/o09-166

[21] Kelley KD, Miller KR, Todd A, Kelley AR, Tuttle R, et al (2010) YPEL3, a p53-regulated gene that induces cellular senes-cence. Cancer Res 70: 3566–3575. 20388804 10.1158/0008-5472.CAN-09-3219 PMC2862112

[22] Hayashi T, Fujita Y, Iwasaki O, Adachi Y, Takahashi K, et al (2004) Mis16 and Mis18 are required for CENP-A loading and histone deacetylation at centromeres. Cell 118: 715–729. 15369671 10.1016/j.cell.2004.09.002

[23] Fujita Y, Hayashi T, Kiyomitsu T, Toyoda Y, Kokubu A, et al (2007) Priming of centromere for CENP-A recruitment by human hMis18alpha, hMis18beta, and M18BP1. Dev Cell 12: 17–30. 17199038 10.1016/j.devcel.2006.11.002

[24] Lee BC, Dikiy A, Kim HY, Gladyshev VN (2009) Functions and evolution of selenoprotein methionine sulfoxide reductases. Biochim Biophys Acta 1790: 1471–1477. 19406207 10.1016/j.bbagen.2009.04.014 PMC3062201

[25] Leung DW, Amarasinghe GK (2012) Structural insights into RNA recognition and activation of RIG-I-like receptors. Curr Opin Struct Biol 22: 297–303. 22560447 10.1016/j.sbi.2012.03.011 PMC3383332

[26] Cui S, Eisenächer K, Kirchhofer A, Brzózka K, Lammens A, et al (2008) The C-terminal regulatory domain is the RNA 5′-triphosphate sensor of RIG-I. Mol Cell 29: 169–179. 18243112 10.1016/j.molcel.2007.10.032

[27] Goenrich M, Bartoschek S, Hagemeier CH, Griesinger C, Vorholt JA (2002) A glutathione-dependent formaldehyde-activating enzyme (Gfa) from Paracoccus denitrificans detected and purified via two-dimensional proton exchange NMR spectroscopy. J Biol Chem 277: 3069–3072. 11741920 10.1074/jbc.C100579200

[28] Burton J, Roberts D, Montaldi M, Novick P, De Camilli P (1993) A mammalian guanine-nucleotide-releasing protein enhances function of yeast secretory protein Sec4. Nature 361: 464–467. 8429887 10.1038/361464a0

[29] Itzen A, Pylypenko O, Goody RS, Alexandrov K, Rak A (2006) Nucleotide exchange via local protein unfolding – structure of Rab8 in complex with MSS4. EMBO J 25: 1445–1455. 16541104 10.1038/sj.emboj.7601044 PMC1440319

[30] Thaw P, Baxter NJ, Hounslow AM, Price C, Waltho JP, et al (2001) Structure of TCTP reveals unexpected relationship with guanine nucleotide-free chaperones. Nat Struct Biol 8: 701–704. 11473261 10.1038/90415

[31] Bommer UA, Thiele BJ (2004) The translationally controlled tumour protein (TCTP). Int J Biochem Cell Biol 36: 379–385. 14687915 10.1016/s1357-2725(03)00213-9

[32] Rehmann H, Brüning M, Berghaus C, Schwarten M, Köhler K, et al (2008) Biochemical characterisation of TCTP questions its function as a guanine nucleotide exchange factor for Rheb. FEBS Lett 582: 3005–3010. 18692051 10.1016/j.febslet.2008.07.057

[33] Shimada T, Hirao K, Kori A, Yamamoto K, Ishihama A (2007) RutR is the uracil/thymine-sensing master regulator of a set of genes for synthesis and degradation of pyrimidines. Mol Microbiol 66: 744–757. 17919280 10.1111/j.1365-2958.2007.05954.x

[34] Han J, Zhang H, Zhang H, Wang Z, Zhou H, Zhang Z (2013) A Cul4 E3 ubiquitin ligase regulates histone hand-off during nucleosome assembly. Cell 155: 817–829. 24209620 10.1016/j.cell.2013.10.014 PMC3994564

[35] Lupas AN, Ponting CP, Russell RB (2001) On the evolution of protein folds: are similar motifs in differ-ent protein folds the result of convergence, insertion, or relics of an ancient pep-tide world? J Struct Biol 134: 191–203. 11551179 10.1006/jsbi.2001.4393

[36] Söding J, Lupas AN (2003) More than the sum of their parts: on the evolution of proteins from peptides. Bioessays 25: 837–846. 12938173 10.1002/bies.10321

[37] Biegert A, Mayer C, Remmert M, Söding J, Lupas AN (2006) The MPI Bioinformatics Toolkit for protein sequence analy-sis. Nucleic Acids Res 34 (Web Server issue) W335–339. 16845021 10.1093/nar/gkl217 PMC1538786

[38] Altschul SF, Madden TL, Schäffer AA, Zhang J, Zhang Z, et al (1997) Gapped BLAST and PSI-BLAST: a new generation of protein database search programs. Nucleic Acids Res 25: 3389–3402. 9254694 10.1093/nar/25.17.3389 PMC146917

[39] Biegert A, Söding J (2009) Sequence context-specific profiles for homology searching. Proc Natl Acad Sci U S A 106: 3770–3775. 19234132 10.1073/pnas.0810767106 PMC2645910

[40] Altschul SF, Gish W, Miller W, Myers EW, Lipman DJ (1990) Basic local alignment search tool. J Mol Biol 215: 403–410. 2231712 10.1016/S0022-2836(05)80360-2

[41] Frickey T, Lupas A (2004) CLANS: a Java application for visualizing protein families based on pairwise similarity. Bioinformatics 20: 3702–3704. 15284097 10.1093/bioinformatics/bth444

[42] Söding J, Biegert A, Lupas AN (2005) The HHpred interactive server for protein homology detection and structure prediction. Nucleic Acids Res 33 (Web Server issue) W244–248. 15980461 10.1093/nar/gki408 PMC1160169

[43] Söding J, Remmert M, Biegert A, Lupas AN (2006) HHsenser: exhaustive transitive profile search using HMM-HMM comparison. Nucleic Acids Res 34 (WebServer issue) W374–378. 16845029 10.1093/nar/gkl195 PMC1538784

[44] Holm L, Rosenström P (2010) Dali server: conservation mapping in 3D. Nucleic Acids Res 38 (Web Server issue) W545–549. 20457744 10.1093/nar/gkq366 PMC2896194

[45] Webb B, Sali A (2014) Protein structure modeling with MODELLER. Methods Mol Biol 1137: 1–15. 24573470 10.1007/978-1-4939-0366-5_1

[46] Guex N, Peitsch MC (1997) SWISS-MODEL and the Swiss-PdbViewer: an environment for comparative protein modeling. Electrophoresis 18: 2714–2723. 9504803 10.1002/elps.1150181505

[47] Deprez C, Lloubes R, Gavioli M, Marion D, Guerlesquin F, et al (2005) Solution structure of the E.coli TolA C-terminal domain reveals conformational changes upon binding to the phage g3p N-terminal domain. J Mol Biol 346: 1047–1057. 15701516 10.1016/j.jmb.2004.12.028

[48] Cock PJ, Antao T, Chang JT, Chapman BA, Cox CJ, Dalke A, Friedberg I, Hamelryck T, Kauff F, Wilczynski B, de Hoon MJ (2009) Biopython: freely available Python tools for computational molecular biology and bioinformatics. Bioinformatics 25: 1422–1423. 19304878 10.1093/bioinformatics/btp163 PMC2682512

[49] Jones E, Oliphant E, Peterson P, et al. (2001) SciPy: Open Source Scientific Tools for Python. Available: http://www.scipy.org/. Accessed 5 Oct 2014.

[50] Hagberg AA, Schult DA, Swart PJ (2008) Exploring network structure, dynamics, and function using NetworkX, in Proceedings of the 7th Python in Science Conference (SciPy2008), Gäel Varoquaux, Travis Vaught, and Jarrod Millman (Eds), (Pasadena, CA USA), pp. 11–5.

