# Supplementary material for: The Thalidomide-Binding Domain of Cereblon Defines the CULT Domain Family and Is a New Member of the β-Tent Fold
Source: PLoS Comput Biol. 2015 Jan 8;11(1):e1004023. doi: 10.1371/journal.pcbi.1004023 (PMC4287342; doi:10.1371/journal.pcbi.1004023)
Supplement: S1 Fig — Comparative models of the β-tent domains in (a) human cereblon and (b) Drosophila yippee. Models are colored according to the templates used (black - CULT domain of M. gryphiswaldense MGR_0879, 4V2Y; red - X. campestris MsrB, 3HCJ; green - human RIG-I, 3EQT). The two central β-hairpins that mount the zinc binding site are shown bold. For each protein, the two following panels show the sequence conservation mapped onto the surface of the model built on the CULT domain of MGR_0879. The red-to-white scale follows highest-to-lowest conservation. Mapping was done using ProtSkin and a multiple sequence alignment derived from two iterations of PSI-Blast and filtered to 70% identity. (DOC) [file pcbi.1004023.s001.doc]

**
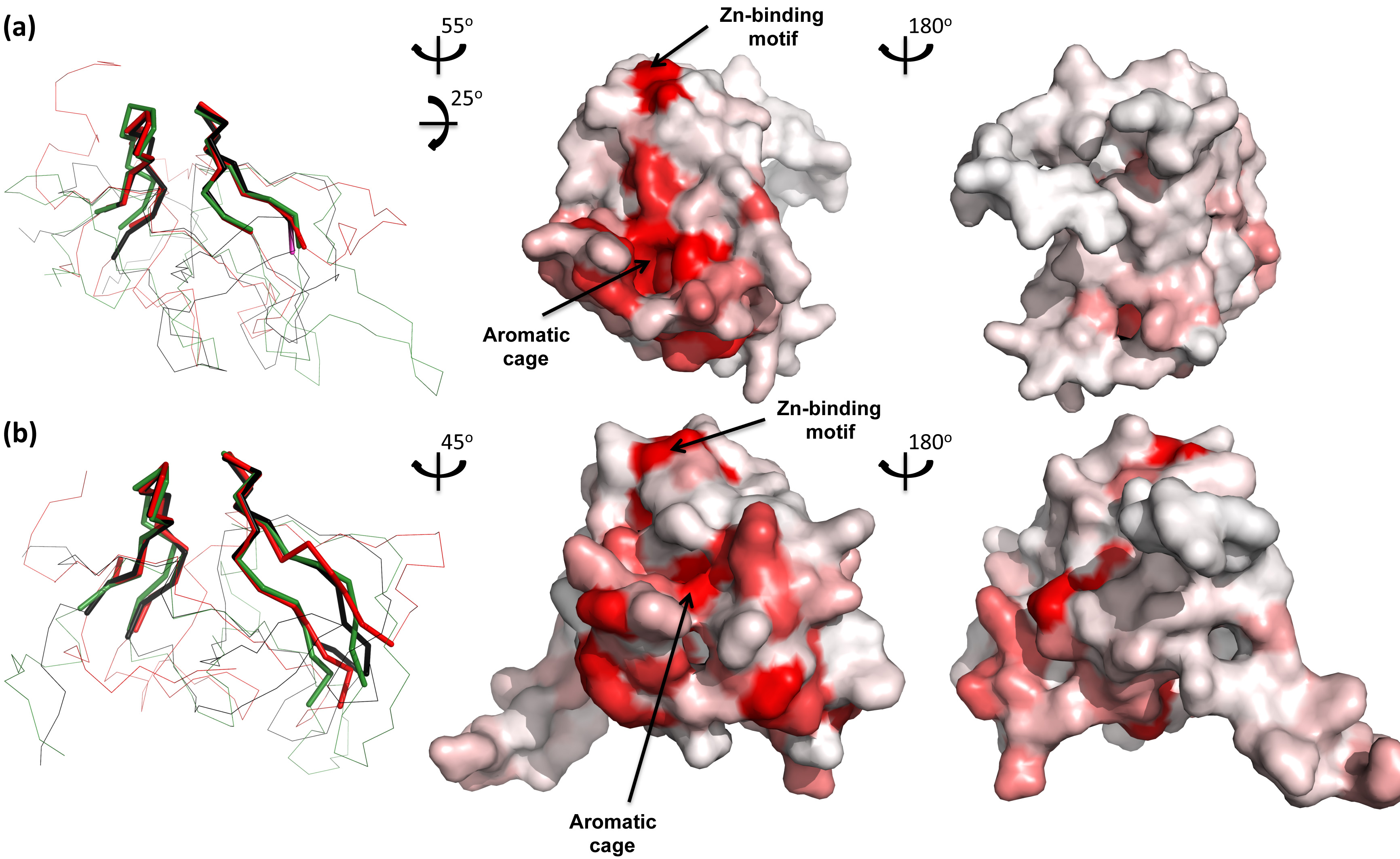
**

**Figure S1. Comparative models of the β-tent domains in (a) human cereblon and (b) Drosophila yippee.** Models are colored according to the templates used (black - CULT domain of *M. gryphiswaldense* MGR_0879, 4V2Y; red - *X. campestris* MsrB, 3HCJ; green - human RIG-I, 3EQT). The two central β-hairpins that mount the zinc binding site are shown bold. For each protein, the two following panels show the sequence conservation mapped onto the surface of the model built on the CULT domain of MGR_0879. The red-to-white scale follows highest-to-lowest conservation. Mapping was done using ProtSkin and a multiple sequence alignment derived from two iterations of PSI-Blast and filtered to 70% identity.
