# Supplementary material for: The Thalidomide-Binding Domain of Cereblon Defines the CULT Domain Family and Is a New Member of the β-Tent Fold
Source: PLoS Comput Biol. 2015 Jan 8;11(1):e1004023. doi: 10.1371/journal.pcbi.1004023 (PMC4287342; doi:10.1371/journal.pcbi.1004023)
Supplement: S2 Fig — Structure gallery of proteins of the β-tent fold superimposed on the crystal structure of M. gryphiswaldense MGR_0879 (grey). The superimposition mainly covers the two four-stranded β-sheets; the β-hairpin insertion that reaches across the bottom of the fold to the C-terminal edge of the second β-sheet is present in all folds, but too struturally variable to superimpose well. A superimposition of the best-conserved structural core of all structures in the figure is shown in the center. The r.m.s.deviations in Cα carbons for the pairwise superimpositions were, in clockwise order: 3HCG: 0.98 Å over 63 residues, 1XA8: 1.72 Å over 30 residues, 3EQT: 1.38 Å over 63 residues, 2FU5: 1.40 Å over 57 resiudes, 1YZ1: 1.31 Å over 50 residues, 3DJM: 1.83 Å over 27 residues. (DOC) [file pcbi.1004023.s002.doc]

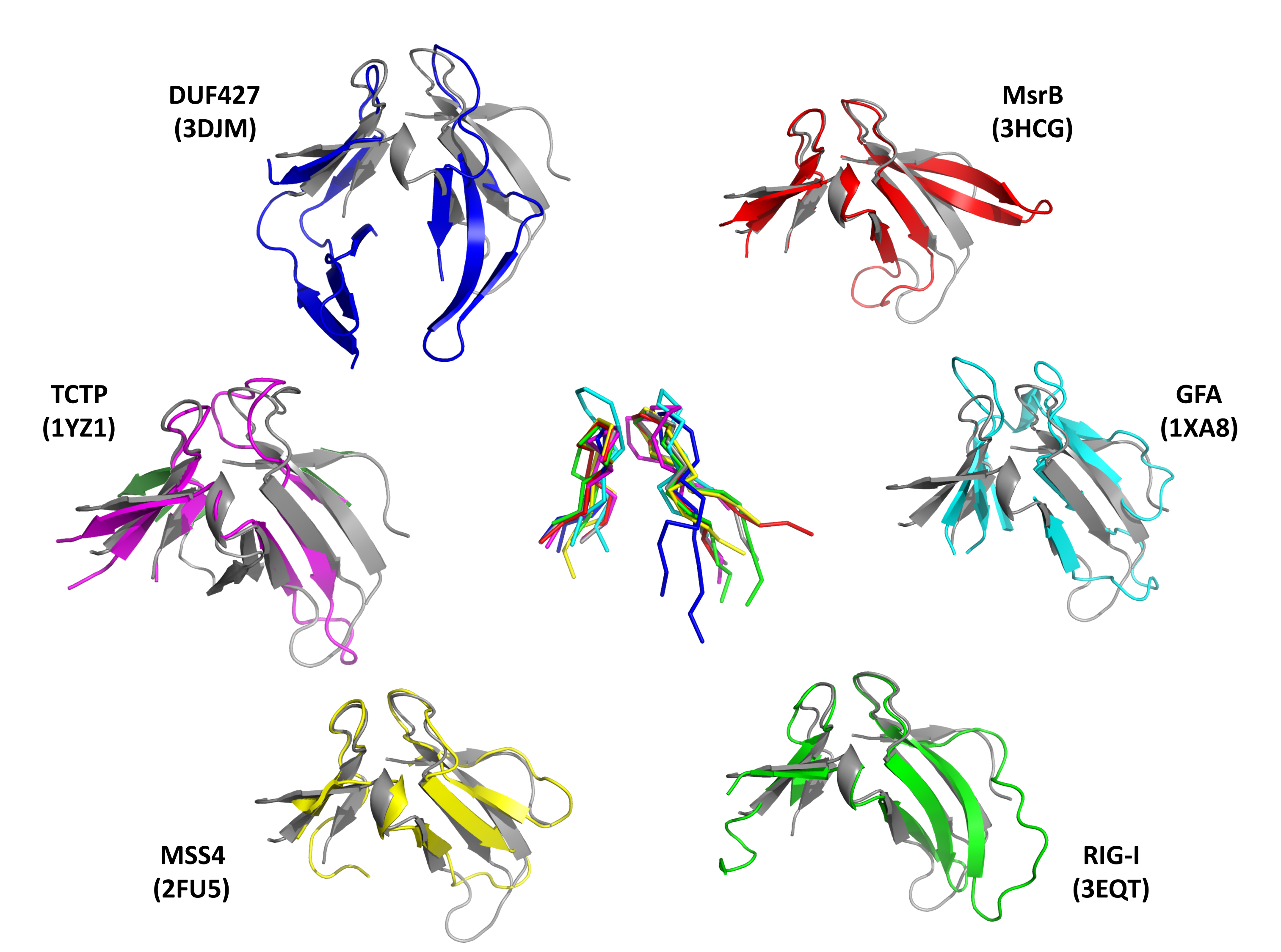


**Figure S2. Structure gallery of proteins of the β-tent fold superimposed on the crystal structure of *M. gryphiswaldense* MGR_0879 (grey).** The superimposition mainly covers the two four-stranded β-sheets; the β-hairpin insertion that reaches across the bottom of the fold to the C-terminal edge of the second β-sheet is present in all folds, but too struturally variable to superimpose well. A superimposition of the best-conserved structural core of all structures in the figure is shown in the center. The r.m.s.deviations in Cα carbons for the pairwise superimpositions were, in clockwise order: 3HCG: 0.98 Å over 63 residues, 1XA8: 1.72 Å over 30 residues, 3EQT: 1.38 Å over 63 residues, 2FU5: 1.40 Å over 57 resiudes, 1YZ1: 1.31 Å over 50 residues, 3DJM: 1.83 Å over 27 residues.
