# Supplementary material for: The Thalidomide-Binding Domain of Cereblon Defines the CULT Domain Family and Is a New Member of the β-Tent Fold
Source: PLoS Comput Biol. 2015 Jan 8;11(1):e1004023. doi: 10.1371/journal.pcbi.1004023 (PMC4287342; doi:10.1371/journal.pcbi.1004023)
Supplement: S3 Fig — Protein-protein association mediated by an aromatic cage. The image shows the main specific contact in the interaction between neuropilin-1 (cyan) and vascular endothelial growth factor-A (VEGF-A) (green), mediated by the docking of the C-terminal arginine of VEGF-A into an aromatic cage formed by neuropilin-1 (PDB: 4DEQ; Parker MW, Xu P, Li X, Vander Kooi CW. Structural basis for selective vascular endothelial growth factor-A (VEGF-A) binding to neuropilin-1 (2012) J Biol Chem 287: 11082–11089). (DOC) [file pcbi.1004023.s003.doc]

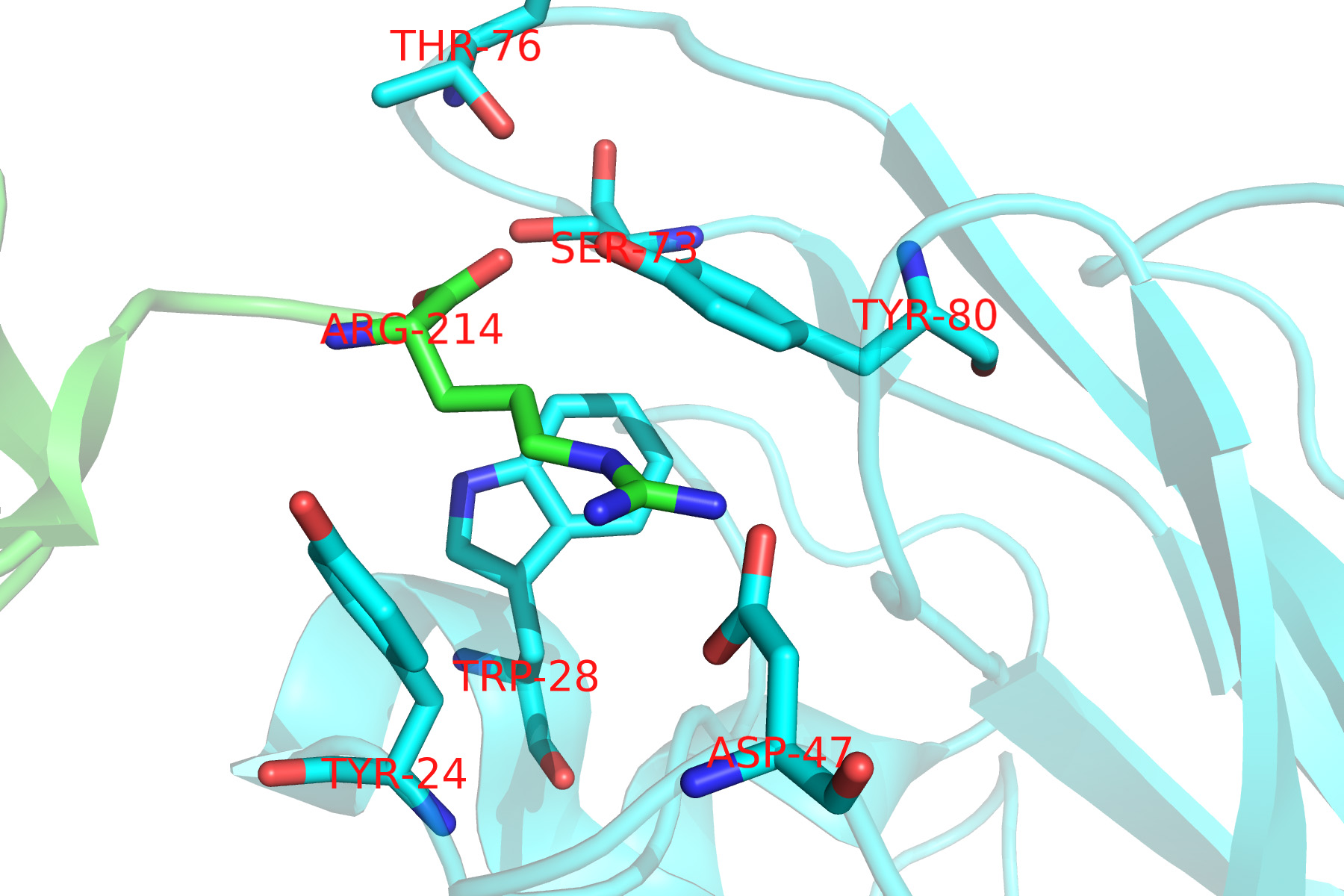


**Figure S3. Protein-protein association mediated by an aromatic cage.** The image shows the main specific contact in the interaction between neuropilin-1 (cyan) and vascular endothelial growth factor-A (VEGF-A) (green), mediated by the docking of the C-terminal arginine of VEGF-A into an aromatic cage formed by neuropilin-1 (PDB: 4DEQ; Parker MW, Xu P, Li X, Vander Kooi CW. Structural basis for selective vascular endothelial growth factor-A (VEGF-A) binding to neuropilin-1 (2012) J Biol Chem 287: 11082-11089).
