# Supplementary material for: The Thalidomide-Binding Domain of Cereblon Defines the CULT Domain Family and Is a New Member of the β-Tent Fold
Source: PLoS Comput Biol. 2015 Jan 8;11(1):e1004023. doi: 10.1371/journal.pcbi.1004023 (PMC4287342; doi:10.1371/journal.pcbi.1004023)
Supplement: S1 Table — Structural similarity of the CULT domains from Magnetospirillum MGR_0879 and human, mouse and chicken cereblon. The numbers show the r.m.s. deviation in Å over the number of superimposed Cα carbons. (DOC) [file pcbi.1004023.s004.doc]

**Table S1.** Structural similarity of the CULT domains from Magnetospirillum MGR_0879 and human, mouse and chicken cereblon. The numbers show the r.m.s. deviation in Å over the number of superimposed Cα carbons.

|  | ***M. gryphiswaldense***  **(4V2Y)** | ***H. sapiens***  **(4TZ4)** | ***M. musculus* (4TZC)** | ***G. gallus***  **(4CI2)** |
| --- | --- | --- | --- | --- |
| ***M. gryphiswaldense*** | - | 0.92/100 | 0.65/83 | 0.78/97 |
| ***H. sapiens* (4TZ4)** | 0.92/100 | - | 0.54/88 | 0.45/108 |
| ***M. musculus* (4TZC)** | 0.65/83 | 0.54/88 | - | 0.49/88 |
| ***G. gallus* (4CI2)** | 0.78/97 | 0.45/108 | 0.49/88 | - |
