# Supplementary material for: The Thalidomide-Binding Domain of Cereblon Defines the CULT Domain Family and Is a New Member of the β-Tent Fold
Source: PLoS Comput Biol. 2015 Jan 8;11(1):e1004023. doi: 10.1371/journal.pcbi.1004023 (PMC4287342; doi:10.1371/journal.pcbi.1004023)
Supplement: S2 Table — Classes of ligands detected in cage-like binding sites in PDB (see also Methods and Fig. 9). (DOC) [file pcbi.1004023.s005.doc]

**Table S2.** Classes of ligands detected in cage-like binding sites in PDB (see also Methods and Figure 9).

| **Group in cage** | **Number** |
| --- | --- |
| Heterocyclic rings | 521 |
| Hydrocarbon rings | 175 |
| Hydrocarbon chains | 119 |
| Hydrocarbon chains containing N/O/S | 110 |
| Tertiary and Quaternary ammonium cations | 45 |
| Others | 82 |
| *Not cage-like* | *46* |
| Total | 1098 |
